# 3D-Printed Gelatin Methacryloyl-Based Scaffolds with Potential Application in Tissue Engineering

**DOI:** 10.3390/polym13050727

**Published:** 2021-02-27

**Authors:** Rebeca Leu Alexa, Horia Iovu, Jana Ghitman, Andrada Serafim, Cristina Stavarache, Maria-Minodora Marin, Raluca Ianchis

**Affiliations:** 1 Advanced Polymer Materials Group, University Politehnica of Bucharest, 1-7 Gh. Polizu Street, 011061 Bucharest, Romania; leurebeca@gmai.com (R.L.A.); jana.ghitman@upb.ro (J.G.); andrada.serafim@gmail.com (A.S.); crisstavarache@gmail.com (C.S.); minodora.marin@ymail.com (M.-M.M.); 2 Academy of Romanian Scientists, 54 Splaiul Independentei, 050094 Bucharest, Romania; 3 “C.D. Nenitescu” Centre of Organic Chemistry, 202-B Spl. Independentei, 060023 Bucharest, Romania; 4 Collagen Department, Division Leather and Footwear Research Institute, National Research and Development Institute for Textile and Leather, 93 Ion Minulescu Str., 031215 Bucharest, Romania; 5 National R-D Institute for Chemistry and Petrochemistry ICECHIM—Bucharest, Spl. Independentei 202, 6th District, P.O. Box 35/174, 060021 Bucharest, Romania; ralumoc@yahoo.com

**Keywords:** biomaterials, hydrogels, gelatin, GelMA, 3D printing, photopolymerization

## Abstract

The development of materials for 3D printing adapted for tissue engineering represents one of the main concerns nowadays. Our aim was to obtain suitable 3D-printed scaffolds based on methacrylated gelatin (GelMA). In this respect, three degrees of GelMA methacrylation, three different concentrations of GelMA (10%, 20%, and 30%), and also two concentrations of photoinitiator (I-2959) (0.5% and 1%) were explored to develop proper GelMA hydrogel ink formulations to be used in the 3D printing process. Afterward, all these GelMA hydrogel-based inks/3D-printed scaffolds were characterized structurally, mechanically, and morphologically. The presence of methacryloyl groups bounded to the surface of GelMA was confirmed by FTIR and ^1^H-NMR analyses. The methacrylation degree influenced the value of the isoelectric point that decreased with the GelMA methacrylation degree. A greater concentration of photoinitiator influenced the hydrophilicity of the polymer as proved using contact angle and swelling studies because of the new bonds resulting after the photocrosslinking stage. According to the mechanical tests, better mechanical properties were obtained in the presence of the 1% initiator. Circular dichroism analyses demonstrated that the secondary structure of gelatin remained unaffected during the methacrylation process, thus being suitable for biological applications.

## 1. Introduction

In order to create biomaterials that could be considered scaffolds for cell culture, which allow cells to proliferate and regenerate the tissue, fundamental cell behavior, specific tissue environment, degradation, and reasons for tissue damage should be well known. The scaffolds designed for tissue engineering should mimic morphological, topographical, and biological properties of extracellular matrix (ECM), because ECM modulates and directs cell behavior. A powerful tool in the research field is represented by the modern technologies that are able to design scaffolds based on biopolymers. These scaffolds can satisfy both the biological and mechanical (supportive) requirement [1].

3D printing is considered the newest method used to design three-dimensional networks with versatile properties. 3D printing is a noninvasive method that allows the use of the material without affecting its vital properties being recommended in tissue engineering.

In order to produce 3D structures, a multitude of bio-inks have been widely studied. To be employed in regenerative medicine, these injectable materials should allow precise control of the tissue architecture for obtaining a personalized implant. Among materials that are studied, hydrogels are the most innovative biomaterials used to print three-dimensional structures in tissue engineering. As printing materials, hydrogels must present a shear thinning behavior, a property that allows continuous flow and printing under a high shear rate [2,3]. In addition, hydrogels possess mandatory criteria in order to recreate the complexity of natural extracellular matrix (ECM) such as biocompatibility and biodegradability, tunable mechanical properties, and precise control of the multi-scale internal architecture of scaffolds [4,5].

Gelatin is one of the most extensively studied proteins for the development of bio-ink formulations used in 3D printing and bioprinting. Gelatin is a protein soluble in water, extracted from animal collagen through partial acid (type A) or alkaline (type B) hydrolysis [6]. Gelatin exhibits an amphoteric character having hydrophilic amino acid sequences like arginine-glycine-aspartic acid (Arg-Gly-Asp) in its structure, and hydrophobic groups in an estimated ratio of 1:1:1. The hydrophobic groups presented in the gelatin structure are leucine, isoleucine, methionine, and valine [6,7,8]. Moreover, gelatin is a biocompatible, nontoxic, and nonimmunogenic polymer, a biomimetic peptide with the ability to prevent cell apoptosis [9,10]. Additionally, cell adhesion is improved by natural cell binding motifs, such as arginine-glycine-aspartic acid peptides from the gelatin structure. Due to these properties, gelatin promotes cell proliferation and differentiation in a certain direction, an essential process for determining the behavior and functions of the tissue, thus accelerating tissue regeneration [9,11,12,13]. Moreover, gelatin resembles the chemical structure and biological functions of collagen in the native ECM. Due to these characteristics, gelatin is considered an ideal material that could emulate the natural structure of the ECM [14,15,16]. For example, gelatin was used to design and fabricate nerve guidance conduits (NGCs) to repair large gap nerve injuries [17]. As it was expected, the results showed that the multichannel NGCs were obtained, and biological analysis proved that NGCs supported the proliferation and migration of neural cells along the channel [17]. In another study, a tri-layered scaffold based on gelatin methacrylate (GelMA)-HA (hydroxyapatite) was designed and fabricated in order to be used for repairing the defects of cartilage and subchondral bone tissue simultaneously. The physical, mechanical, and biological analysis showed that the properties of the scaffold obtained are appropriate for repairing the defects of the subchondral bone tissues simultaneously [18]. Bektas and Hasirci designed a 3D-bioprinted corneal stroma-equivalent in order to substitute the native tissue, and the study results showed that GelMA hydrogels bioprinted with keratocytes presents excellent transparency, adequate mechanical strength, and high cell viability, so the obtained hydrogel imitated the biological and physical properties of the corneal stroma [19].

Gelatin is a natural protein with low mechanical and thermal properties (at 37 °C, it becomes solution due to the cleavage of the hydrogen bonds), degrades quickly, and has no mechanical stability in the long term [20,21], which represents a drawback in the development of a biomaterial intended for medical applications. However, both mechanical and thermal features of gelatin can be improved by crosslinking [22]. Gelatin exhibits a folding structure that can be easily modified by crosslinking its functional groups with formaldehyde, acetic acid, potassium iodide [23], or with different targeting ligands, -thiol groups, cationic agents, alendronate, PEG, and conjugating targeting EGFR peptide [11].

Because gelatin can become toxic after crosslinking with toxic compounds, in order to obtain a material usable in tissue engineering, gelatin was modified with methacrylic anhydride. Methacrylic anhydride reacts with amino groups from the side chains of gelatin. The resulting methacrylated gelatin named GelMA by van Den Bulcke et al. [21] is similar to gelatin because its properties like arginine–glycine–aspartic acid (RGD) sequences, biocompatibility, degradation properties, and its transition influenced by temperature are maintained [21]. Moreover, methacrylate groups can be used to make gelatin easily polymerizable into a hydrogel that is stable at 37 °C.

The main target of our study was to design a 3D scaffold based on gelatin methacrylate (GelMA) with potential application in tissue engineering. In this respect, gelatin with different methacrylation degrees was synthesized to obtain hydrogel materials with optimal properties to be used in printing. GelMA was synthesized by binding the methacrylic groups on the gelatin surface through a covalent reaction. Due to the grafted methacryloyl groups, the biopolymer can be crosslinked by a versatile photocuring process using biocompatible photoinitiators (TiO_2,_ LAP, Irgacure 2959) [8]. Photopolymerization using UV light provides good temporal and spatial control over the crosslinking mechanism, in order to attain a 3D scaffold with unique properties at room temperature. Besides, several studies demonstrated that the functions and viability of the cells were not compromised by the photopolymerization process [8,24,25].

In addition, gelatin can be thermally crosslinked at low- or high-temperature conditions, with consequences in the gelatin structure. It is worth mentioning that the thermal crosslinking process is a reversible process based only on physical interaction. However, photopolymerization using UV light provides many advantages over the thermal and chemical crosslinking process; namely, the crosslinking process takes place quickly at room temperature, and provides thermal stability and mechanical stability over time.

GelMA-based hydrogels exhibit high morphological and mechanical stability with tunable mechanical properties, inherent bioactivity, and physicochemical tailorability and allow cells to attach and spread in the GelMA scaffold and proliferate and regenerate tissue. In addition, hydrogels based on GelMA could act as a platform allowing the control of cell behavior [25,26,27].

Unlike other studies related to GelMA printing [17,18,19], the present work is a systematic study developed to synthesize appropriate GelMA hydrogel ink formulations suitable to be functional in the 3D printing process. Therefore, three methacrylation degrees of GelMA, three different concentrations of GelMA (10%, 20%, and 30%), and also two concentrations of photoinitiator (I-2959) (0.5% and 1%) were explored. Subsequently, in order to demonstrate the obtaining of methacrylated gelatin, as well as to characterize it, several analyses were conducted.

The GelMA precursor solution and, further, its crosslinked formulations were characterized by ^1^H-NMR-spectrometry, Fourier-transform infrared spectrometry (ATR-FTIR) for structural characterization, circular dichroism (DC), dynamic light scattering (DLS) to determine the isoelectric point, the contact angle, swelling behavior to determine the hydrophilicity, printability for setting the printing parameters of the injectable formulations, and nanoindentation for testing the mechanical properties. In addition, in order to observe how the methacrylation degree and photoinitiator concentration affect the mechanical properties, the crosslinking density was calculated by rheology [28,29]. Finally, electronic microscopy (SEM) was used to determine the morphology of the obtained materials [30]. Statistical analyses were performed in order to study if differences between the obtained hydrogel inks were significant.

## 2. Materials and Methods

### 2.1. Materials

Gelatin (from bovine skin, gel strength ~225 g Bloom, type B) (Sigma-Aldrich, St. Louis, MO, USA), methacrylic anhydride (MA) (MW = 154.16 g/mol) (Sigma-Aldrich, Goettingen, Germany) and 2-hydroxy-4′-(2hydroxyethoxy)-2-methylpropiophenone (Irgacure 2959 (I-2959), (MW = 224.25 g/mol; d = 1.035 g/mL)) was purchased from Sigma-Aldrich, Milano, Italy; ultrapure water and phosphate-buffered saline (PBS) solution pH = 7.4, were prepared in our laboratory.

### 2.2. Experimental

Functionalized gelatin (GelMA) was obtained according to the previously reported method [7,25]. The degree of methacrylation was controlled by the amount of methacrylic anhydride added (Table 1). Briefly, 5 g of gelatin was dissolved in 45 mL PBS (pH = 7.4) at 50 °C for 1.5 h until total solubilization. Then, depending on the degree of methacrylation, different amounts of methacrylic anhydride were added to the gelatin solution. Afterward, in order to allow methacrylic anhydride to react with amino functional groups from the protein structure, the reaction was maintained at 50 °C for 2 h under vigorous magnetic stirring. The obtained solution was dialyzed against distilled water using dialysis cellulose bags (MWO = 1200 Da) for 72 h. Functionalized gelatin was lyophilized at 0.28 bar for 72 h (D-37520,CHRIST, Osterode am Harz, Germany) and stored at room temperature.

### 2.3. Preparation of the GelMA-Based Inks

To determine the optimal compositional mixture for the 3D printing process, the precursor solutions were prepared by totally dissolving GelMA (10 or 20% w/v%) with different methacrylation degrees in PBS (pH = 7.4) at 40 °C, under gentile stirring. Then, to the obtained GelMA solution, the photoinitiator (I-2959) (Sigma-Aldrich, Milano, Italy) was added in a concentration of 0.5% or 1% w/v% from the total amount of polymer (Table 2). The mixture was subjected to homogenization by magnetic stirring until the photoinitiator was completely dissolved, in order to obtain a perfectly homogeneous mixture.

### 2.4. ^1^H-NMR-Spectrometry of Gelatin and Gelatin Methacrylate (GelMA)

The ^1^H-NMR spectrometry was used to determine the methacrylation degree of GelMA. In order to make the analyses, 20 mg GelMA was dissolved in 0.75 mL D_2_O to clear solution. The spectrum was registered on a Bruker NMR 600 MHz Advance spectrometer (Bruker BioSpin, Rheinstetten, Germany). The methacrylation degree was calculated using Equation (1):

(1) $$Methacrylation\;degree\;\left(\%\right)=1-\frac{\;integration\;signal\;of\;arginine\;from\;GelMA}{\;integration\;signal\;of\;arginine\;from\;Gelatin}\times 100$$

### 2.5. Fourier-Transform Infrared Spectrometry (ATR-FTIR)

The structural characterization of raw material and GelMA with different methacrylation degrees was performed using ATR-FTIR spectrometry. FTIR spectra were recorded on a Vertex 70 Bruker FTIR spectrometer (Bruker, Billerica, MA, USA) equipped with an attenuated total reflectance (ATR) accessory. For all the formulations, the FTIR spectra were registered in the ATR mode, at a resolution of 4 cm^−1^ in the 600–4000 cm^−1^ wavenumber region.

### 2.6. Determination of the Isoelectric Point (IP)

Isoelectric points (IPs) of gelatin and GelMA with different degrees of methacrylation were determined by the DLS technique, monitoring the variations in zeta potential of samples in different pH media. Thus, different solutions consisting of a 1 mg/mL concentration of gelatin, GelMA 1.5%, GelMA 2.5%, and GelMA 5% in saline solution (1 mM NaCl) were subjected to analysis. Samples were introduced in a ZETASIZER instrument (Zetasizer Nano ZS Malvern Instrument, Worcestershire, UK) and were measured at different pH values, ranging from 2 to 8, at 25 °C in three cycles, each cycle with 50 measurements. All the measurements were performed in triplicate.

### 2.7. Circular Dichroism (CD)

Circular dichroism counts on the differential absorption of left-right-polarized circular light of a chiral macromolecule. Spectroscopy acquisition was performed on a Jasco J-1500 Spectrophotometer, Tokyo, Japan (J-1500 Circular Dichroism Spectrophotometer) using a quartz cell with l = 1 mm. For each reading, 500 μL of functionalized/nonfunctional gelatin aqueous solution was placed in a cuvette at a concentration of 0.005% and the spectra were registered by scans in triplicate, using a spectral range of 176–250 nm, at a scan rate of 100 nm/min. The pH value at which the molecule has no electric charge is determined by PI, due to the equal concentration of negatively and positively charged molecular species that are present in the protein solution. The pH value at which Zeta Potential (ZP) is zero was considered the PI of the protein.

### 2.8. GelMA-Based Hydrogels Morphology, Printability, and Scanning Electron Microscopy (SEM) Analyses

In order to characterize the printability of the hydrogel materials, a series of tests were performed using the direct dispensing print head of the bioprinter 3DDiscovery™ from RegenHU Ltd., Villaz-St-Pierre, Switzerland. Tests were repeated five times in order to verify the reproducibility of the selected method and the stability of the 3D-printed hydrogel. A syringe of 3 mL having attached a cylindrical nozzle of 25 G (needle gauge ø 0.25 mm, needle length 6.35 mm) or 23 G (ø 0.33 mm, needle length 6.35 mm) was used. Different printing speeds ranging from 5 to 10 mm/s, and pressures in the range of 150–300 kPa were tested. All the samples were printed at room temperature. For the scaffold architecture, the special BIOCAD software (RegenHU Ltd., Villaz-St-Pierre, Switzerland) was selected. This software allows the drawing in 2D of objects that can be viewed in 3D after generating the G code. Scaffolds were printed layer by layer in 20 layers.

In order to perform SEM analysis, the obtained scaffolds were lyophilized and broken manually. The broken samples were analyzed using environmental scanning electron microscopy (ESEM-FEI Quanta 200, Eindhoven, The Netherlands) and the samples were analyzed as such, without being sputter-coated. In addition, in order to obtain the secondary electrons images, a gaseous secondary electron detector (GSED) detector was used in the following conditions: 25–30 kV accelerating voltage using magnifications in the range of 2000–5000× for fracture surface of GelMA samples, and a pressure of 2 torr (vacuum conditions).

### 2.9. Swelling, Dissolvability, and Contact Angle Measurements of the 3D-Printed Hydrogel Based on GelMA

The swelling ratios of hydrogels were measured at predetermined periods of time by determining the weight of each sample after removing the residual liquid [30,31,32,33].

The degree of swelling was calculated using Equation (2):

(2) $$\mathrm{Degree}\;\mathrm{of}\;\mathrm{swelling}\;(\%)\;=\;\frac{Ww-Wd}{Wd}*100$$

where: *W_w_* = wet weight, *W_d_* = dry weight.

In order to study the dissolvability of the 3D-printed biomaterials, hydrogel-based lyophilized discs were immersed in PBS for 24 h and then subjected to weighting. Before each weighting, the residual liquid from the discs was removed [33,34].

Dissolvability was calculated using Equation (3):

(3) $$\mathrm{Dissolvability}\;(\%)\;=\;\frac{Wo-Wd}{W0}*100$$

where: *W_o_* = initial weight, *W_d_* = dry weight.

The hydrophilicity of the crosslinked 3D-printed scaffolds based on GelMA was determined by measuring their water contact angle. The water contact angle was determined using the Drop shape analyzer DSA100 (KRUSSS, 22453 Hamburg, Germany) equipped with a CCD video camera. A specific amount of each tested sample was attached on a glass slide and was then placed in the sample stage. The behavior of the droplet was measured, and the water contact angles were provided by software automatic calculation.

### 2.10. Mechanical Properties and Rheological Tests

The effect of the methacrylation degree of GelMA on the mechanical properties of the crosslinked 3D-bioprinted hydrogels was studied by the nanoindentation technique. All analyses were performed on a Nano Indenter^®^ G200 (Santa Clara, CA, USA), and an Espress Test to a Displacement was taken. This method allows determining very quickly the Young’s modulus and the hardness [34]. The principle of the indentation test is described as follows: The sample is moved by the system from the microscope to the XP head when the test is initiated. After that, all indents are performed in rapid succession; for each indentation, the sample is moved automatically under the Indenter tip. When all the analyses are complete, the head disengages, and the sample is moved by the system under the microscope [34].

Storage and loss moduli (G’ and G”, respectively) were measured using a Kinexus Pro Rheometer (Malvern, Worcestershire, UK) equipped with a Peltier element for precise temperature control and 20 mm parallel-plate geometry. The results were registered in which the gap was maintained at a normal force of 0.2 N. The linear viscous region was established and the frequency sweep measurements were performed at a stress of 5 Pa for all samples within the frequency range of 0.01–10 Hz. The crosslinking density was computed using Equation (4) [30], where G’ is the storage modulus, R represents the gas constant, and T the temperature at which G’ was measured (298.15 K).

(4) $$\upsilon \;=\frac{G^{\prime }}{RT}$$

### 2.11. Statistical Analyses

The data are expressed as mean ± SD. The significance of differences was evaluated by single-factor ANOVA test and it was considered significant if *p* < 0.05.

## 3. Results and Discussion

**Figure 1:**
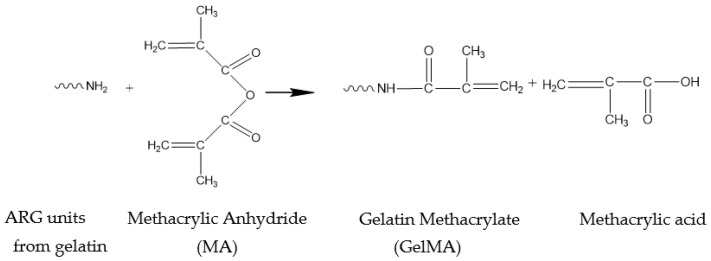
Methacrylation of gelatin.

GelMA was synthesized by binding the methacrylate groups on the gelatin surface through a covalent reaction with the amine groups from the protein (Figure 1). Afterward, the obtained GelMA was subjected to photocuring in order to obtain a crosslinked 3D scaffold.

### 3.1. ^1^H-NMR

**Figure 2:**
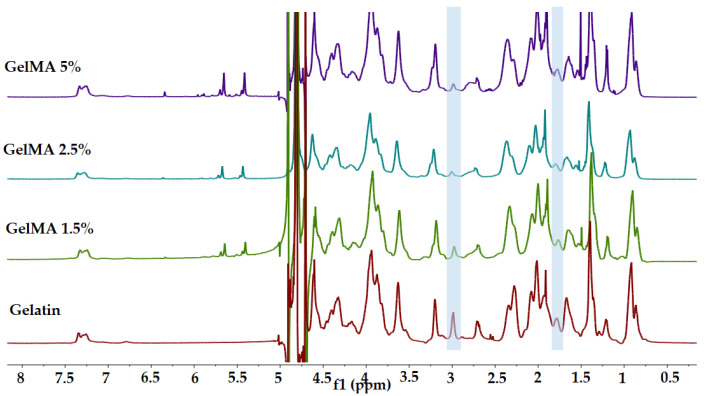
^1^H-NMR spectra of gelatin, GelMA 1.5%, 2.5%, 5%.

The methacrylation degree of GelMA was determined by ^1^H-NMR spectrometry. ^1^H-NMR spectra registered for all analyzed samples are presented in Figure 2. Methacrylate functional groups were grafted onto the gelatin backbone through the reaction between methacrylic anhydride and arginine residues units. In this context, the modification of arginine residues units with methacrylate groups was confirmed by the decrease in the arginine (2H) signal at 2.9 ppm, and by the increase in the methyl group (3H) signal at 1.8 ppm (in all spectra recorded for GelMA) [33,35].

Additionally, GelMA-registered spectra revealed the presence of two new signals at δ = 5.4 and 5.6 ppm corresponding to the acrylic protons (2H) of methacrylic functions characteristic to the MA structure that was grafted onto the gelatin backbone and led to the synthesis of GelMA. It is also important to mention that the area of these signals increases with MA concentration, indicating a high degree of methacrylation (methacrylation degree of GelMA with 1.5%, 2.5%, and 5% MA was 63%, 64%, and 66% respectively).

In order to normalize the amine signals (2.9 ppm) of methacrylated arginine, the phenylalanine signals (7.0–7.5 ppm) for five protons were set as an internal reference. Among all the samples, the internal reference was not modified by reaction with methacrylic anhydride [35].

### 3.2. ATR-FTIR

**Figure 3:**
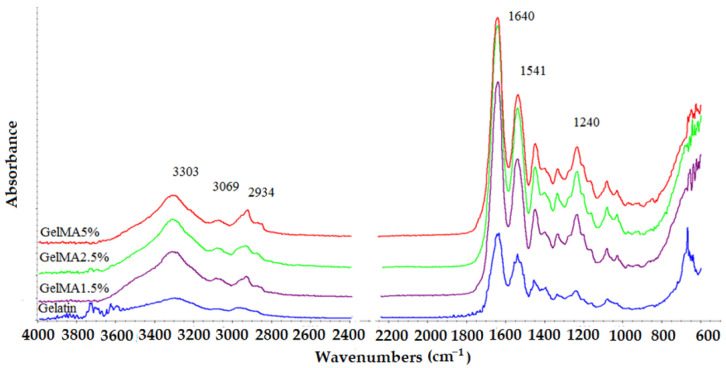
FTIR spectra of gelatin, GelMA1.5%; GelMA2.5%; GelMA5%.

The ATR-FTIR spectra recorded for the obtained samples are presented in Figure 3.

The characteristic absorption bands of functional groups from the gelatin structure are also found in the FTIR spectra recorded for methacrylated GelMA samples, being positioned at the same wavelength. The spectra registered for all the xerogels based on gelatin and GelMA with different degrees of methacrylation exhibit a peak at 3290 cm^−1^ related to the stretching vibration of OH groups from the hydroxyproline units [33,34]. The band from 3200 to 3400 cm^−1^ represents the presence of the peptide bond (N-H stretching) from amide A, as well as the signal at 3069 cm^−1^ attributed to the stretching vibration of the C-H bond from amide B, which was also registered in all the spectra [33,34].

The GelMA spectrum showed peaks at 1640, 1541, and 1240 cm^−1^ related to the C=O stretching (amide I), N-H bending (amide II), and C-N stretching plus N-H bending (amide III) [33,34], respectively. Moreover, a N-H stretching (amide A) could be observed at 3303 cm^−1^, as well as a stretching vibration of CH at 2934 cm^−1^ corresponding to the symmetric and asymmetric stretching in the CH_2_ groups of alkyl chains (Figure 3) [31,32,33,35,36].

### 3.3. Isoelectric Point

The IP of gelatin and GelMA with different methacrylation degrees was determined by measuring the zeta potential of each sample at various pH values.

**Figure 4:**
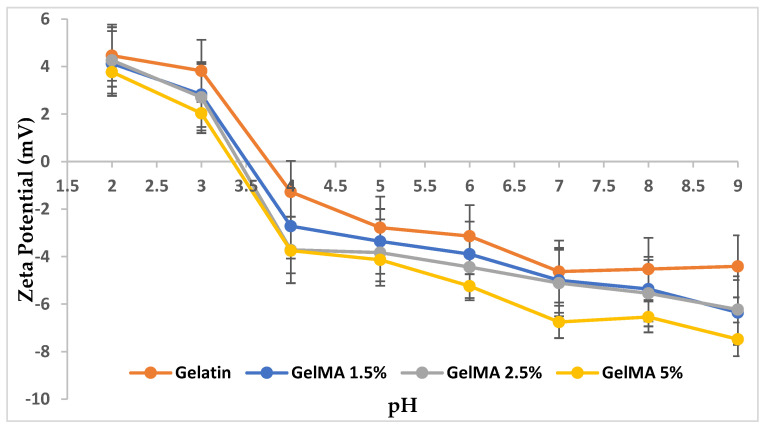
The isoelectric point (IP) of all analyzed samples.

The results disclosed in Figure 4 pointed out that as the degree of methacrylation reaction increases, the value of the isoelectric point decreases.

The functionalization reaction of the gelatin with MA took place between amino (NH_2_) groups from arginine units in gelatin and methacrylic groups from MA. Therefore, the increase in the MA concentration in the methacrylation reaction involved a high number of amino and hydroxyl groups causing their consumption. The excess of the unreacted carboxyl groups from the MA structure respectively provided an acidic character and a low value of IP, which was shifted to lower pH values with the methacrylation degree.

### 3.4. Circular Dichroism

Circular dichroism (CD) is a method that provides the possibility to study the conformations adopted by proteins and nucleic acids in solution. This technique allows the determination of any structural alterations that might result from changes in environmental conditions, such as pH, temperature, and ionic strength [37,38,39]. CD is usually employed to analyze the secondary structure of the proteins [40].

**Figure 5:**
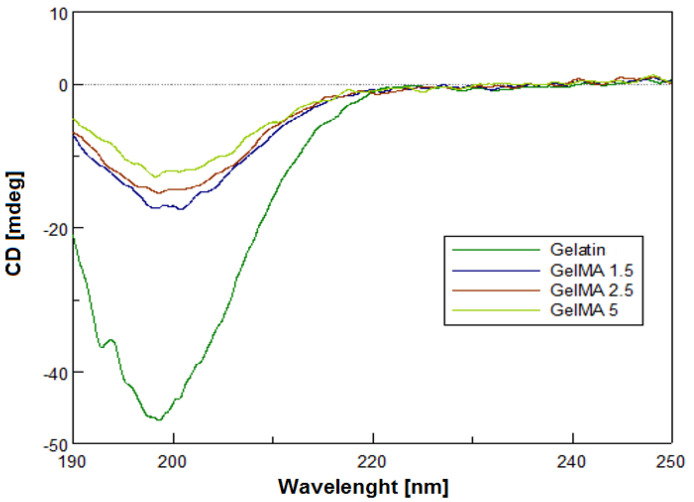
Rough overlapping CD spectra (190–250) of gelatin and GelMA samples.

The GelMA structure was studied in order to observe if the secondary structure of gelatin was preserved during the methacrylation reaction and if the methacryloyl functionalization of GelMA interfered with helix formation. The CD spectra registered for the obtained samples are presented in Figure 5.

CD spectra of GelMA with different degrees of methacrylation indicated a negative band around 199–201 nm when compared to the gelatin spectra, which is the characteristic band of a structure with random coils and β-sheets (structures formed by the binding of two or more B-chains adjacent to each other via hydrogen bonds) specific to the secondary gelatin structure [41]. Functionalized samples showed a negative peak amplitude at 198 nm (the higher the degree of methacrylation, the lower the ellipticity). This decrease may be caused by methacrylated pendant groups as noticed by other research groups [42]. The intensity of the negative band of the spectrum registered for GelMA with high methacrylation degree ascribed to a portion of random coil formation was slightly higher than that of GelMA with a low methacrylation degree. Therefore, the growth of the methacryloyl functionalization of GelMA brought out random coil formation. In addition, along with the increase in the degree of metacrylation of gelatin, the triple-helix contents of GelMA at 220 nm decreased. The intrachain or interchain hydrogen binding in the triple helix could be reduced by the methacrylation of hydroxyl or amino groups from the gelatin chain. Thus, with the increase in the degree of methacrylation, the random coil increases [43].

### 3.5. Printability and Morphology of GelMA Hydrogels

**Figure 6:**
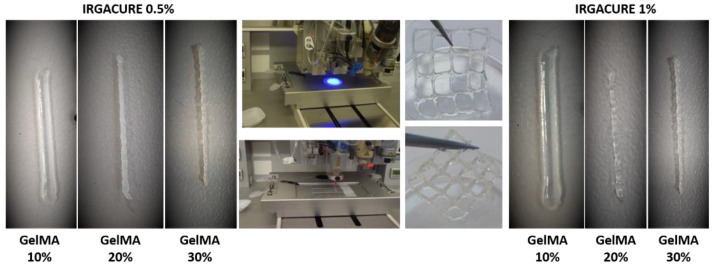
3D printing process and printing tests.

The main focus of this work was to develop a printable GelMA-based hydrogel ink able to mimic the biological and functional complexity of native tissues. The photoinitiator selected for our study was Irgacure 2959 (I-2959). Compared with other photoinitiators, Irgacure 2959 is low-cytotoxicity and, under UV-light, dissociates into benzoyl and ketyl free radicals [44]. In this respect, we followed the development of different hydrogel inks using several concentrations of GelMA but also photoinitiator (I-2959) (Table 2). The 3D printing process was performed at room temperature, and the photocrosslinking step was achieved under the UV light (365 nm with a maximal optical output power of 500 mW)) for 5 min, at a distance of 5 cm from the sample (Figure 6). Different needle materials (stainless steel and polyethylene) and needle dimensions (0.25 mm, 0.33 mm) were adjusted to obtain 3D constructions through extrusion technology.

In order to study the effect of the precursor concentration on the printability properties and on the final characteristics of the 3D-printed hydrogels, three concentrations of GelMA solution were prepared and subjected to printing tests.

First, hydrogels with 10% GelMA and 0.5% or 1% I-2959 were prepared and subjected to the additive manufacturing process. The low concentration of GelMA leads to low viscosities of the bio-inks inducing instability during the extrusion process; therefore, irregular shapes of the filament were obtained. In addition, the gelatin filaments were larger and spread more than GelMA 20% and GelMA 30% after they came into contact with the glass slides surface on which they were printed.

Regardless of the concentration of photocrosslinker agent used, the structural integrity of the filaments was not maintained, due to the slow gelation time of the ink, and the poor mechanical properties did not allow the scaffolds to maintain their initial structural integrity. When the GelMA concentration in the precursor solution was increased to 20%, as expected, the apparent viscosity of the inks increased and allowed the printing and obtaining of stable filaments shapes, without spreading on the glass slides. After printing, the scaffolds were exposed to UV light and preserved their integrity and geometry. Further, in order to select the optimal polymer concentration, the concentration of GelMA was increased more to 30% in the initial printing ink. The result was an apparent viscous ink that impeded the printing process and led to wrinkled filaments (Figure 6).

According to the obtained results, the 3D hydrogels consisting of GelMA 20% with 0.5% I-2959 and 1% I-2959 presented the optimal characteristics in terms of filaments integrity and geometry shape stability of the printed constructs. These formulations were selected for further investigation in order to obtain a material intended for tissue regeneration [4,45].

Finally, a scaffold with a square shape and grids (2 × 2 cm), with 20 layers, was designed in BIOCAD and printed. The resulting scaffold presents homogeneity and stability when photo-cured for 5 min using UV light (365 nm with a maximal optical output power of 500 mW).

In order to observe the morphology of nonmineralized GelMA hydrogels and the morphology of GelMA hydrogels after mineralization, a sample with a concentration of 20% GelMA and 1% photoinitiator was subjected to SEM analyses.

**Figure 7:**
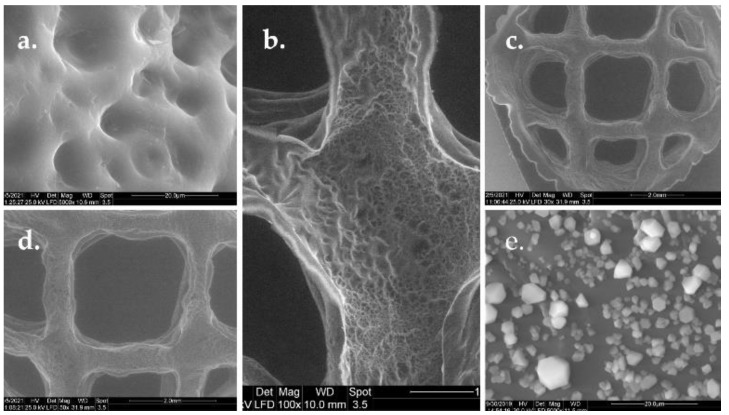
SEM images showing the microstructure aspect of: (**a**) Nonmineralized GelMA scaffold at mag. 5000×, (**b**) 100×, (**c**) 30×, and (**d**) 50×; (**e**) mineralized GelMA scaffold at mag. 5000× with scale bar of 20 μm.

The sample presented high porosities and interconnected macropores before mineralization (Figure 7). These macropores are separated by smooth thin-surface walls. Additionally, the walls of scaffolds presented a highly porous structure. The pores showed different sizes and were obtained during the photopolymerization process. The syneresis phenomenon prevents the system from developing a homogenous material.

One of the most important parameters for biopolymers use in tissue engineering is the roughness of the surface, because it affects the cell behavior—allows the cells to attach, spread, and proliferate in the GelMA scaffold. The in vitro behavior of 3D materials was studied after the mineralization of the hydrogel. Lyophilized hydrogel disks were immersed in PBS at 37 °C. After 5 days, the discs were washed, dried by lyophilization, and stored at room temperature. SEM images performed on the obtained discs revealed that the GelMA mineralized samples retained their 3D microstructure and were covered with spherical agglomerates of minerals. Thus, a good adherence of the material was observed.

### 3.6. Swelling Behavior Studies and Contact Angle Measurements

An important feature of hydrogels for printing is their hydrophilic/hydrophobic character and their susceptibility to degrade by hydrolysis. These characteristics are measured through the swelling ratio. The degree of swelling is influenced by the pore size of a polymer network, concentration of GelMA solution, the methacrylation degree of GelMA, and the amount of photoinitiator used in the crosslinking step. In order to study the swelling behavior of obtained samples, a hydrogel based on GelMA with 58% methacrylation degree was chosen. Samples were prepared from different concentrations of GelMA (10%, 20%, and 30%) using 0.5% and 1% I-2959 against the amount of polymer.

**Figure 8:**
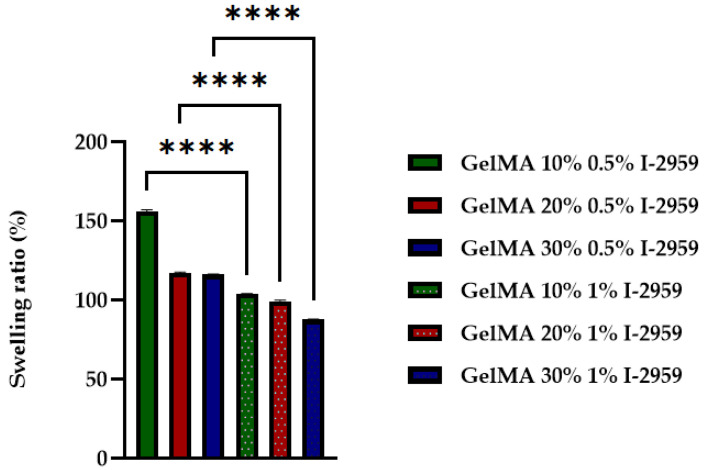
Swelling ratio of GelMA-based hydrogel with different concentrations of GelMA and I-2959. Statistical significance: **** *p* < 0.0001.

**Figure 9:**
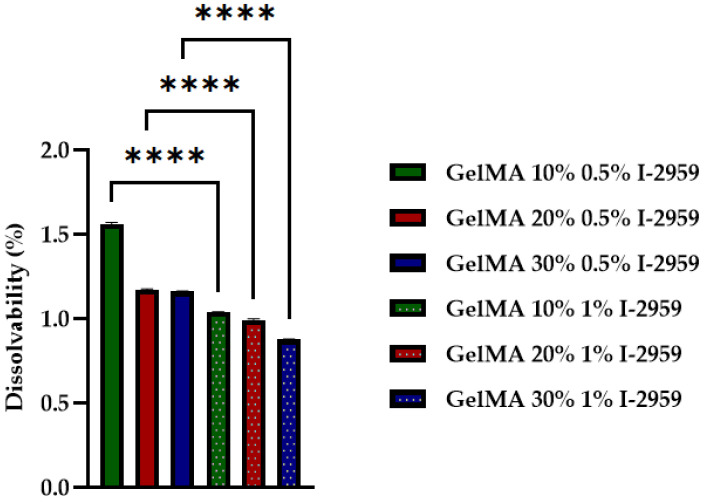
Dissolvability of GelMA-based hydrogel with different concentrations of GelMA and I-2959. Statistical significance: **** *p* < 0.0001.

The crosslinked hydrogels based on GelMA were analyzed in terms of the swelling and degree of dissolvability (Figure 8 and Figure 9).

The capacity of hydrogels to absorb water was influenced by the concentration of the crosslinking agent and the concentration of GelMA used in the synthesis process. Therefore, the swelling degree decreased with the GelMA and initiator concentration. The polymer chains become closer due to the new bond formation after crosslinking, making the mesh denser, with higher retraction forces [45]. The hydrophilicity of the materials increases with the decrease in GelMA concentration, and also with the decrease in photo initiator concentration.

The dissolvability tests of the hydrogels performed in PBS at 37 °C (Figure 8) proved that the stability of the material increased when the photoinitiator was added because of the solid bonds resulted after UV curing.

Thus, with the increase in GelMA and photocrosslinker concentration, a more compacted structure was obtained. The hydrophilicity of the 3D-printed hydrogel decreased, and swelling degree and dissolvability properties also decreased.

**Figure 10:**
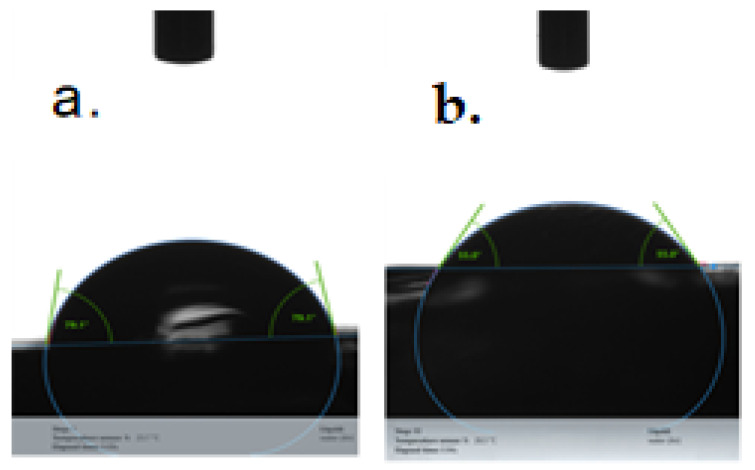
Water contact angle on the samples based on: (**a**) GelMA20% + 0.5% I-2959 and (**b**) GelMA 20% + 1% I-2959.

These results are consistent with other studies that have shown that the swelling degree decreased with the GelMA and initiator concentration [45]. All these results have a statistical significance presented in Figure 9 and Figure 10.

The hydrophilicity of 3D-printed scaffolds based on GelMA was studied by means of the contact angle. To evaluate the effect of the crosslinking agent upon the hydrophilicity of scaffolds, two samples with different amounts of I-2959 (0.5, 1%) were subjected to analysis.

Both GelMA-based scaffolds presented a hydrophilic character, regardless of the amount of photoinitiator, namely: 55.45° ± 0.4 for GelMA20% + 0.5% I-2959 and 78.84° ± 0.27 for GelMA 20% + 1% I-2959) (Figure 10). However, the hydrophilicity of the biomaterial decreased with the concentration of the photoinitiator. This effect can be explained by the presence in the system of a high number of centers that initiate the photocuring process, determining the formation of a denser crosslinking network, and a less hydrophilic system.

### 3.7. Mechanical Properties and Rheological Tests

**Figure 11:**
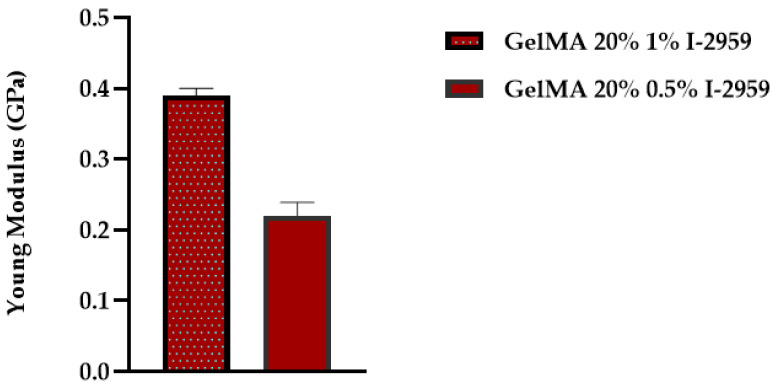
Modulus of GelMA with different concentrations of photoinitiator.

There are several factors that influence the mechanical properties of GelMA, specifically: Degree of methacrylation, the precursor concentration, concentration of the photocrosslinker used, and UV exposure time [46]. Our study focused only on the influence of the initiator over the mechanical properties of GelMA hydrogels. Thus, samples obtained with 20% GelMA and 0.5% or 1% I-2959 were analyzed. The method used to evaluate the materials was “Express Test to a Displacement”. In order to conduct the analyses, an array of 100 indents was performed, to a specified depth of 200 nm and Poisson ratio of 0.4, using the XP head and Berkovich indenter. The results are presented as the average of Young’s modulus for all one hundred indents performed. In order to calculate the Young’s modulus from force–displacement curves, the Oliver Pharr method was used. Figure 11 summarizes the results obtained for Young’s modulus (E).

The results proved that the concentration of the photo initiator is of great importance when we target the improvement in mechanical properties of a material. A value of E = 0.393 ± 0.01 GPa was obtained for GelMA with 1% I-2959, while the value of Young’s modulus determined for GelMA hydrogels with 0.5% I-2959 was approximately 45% lower (E = 0.22 ± 0.019 GPa).

**Figure 12:**
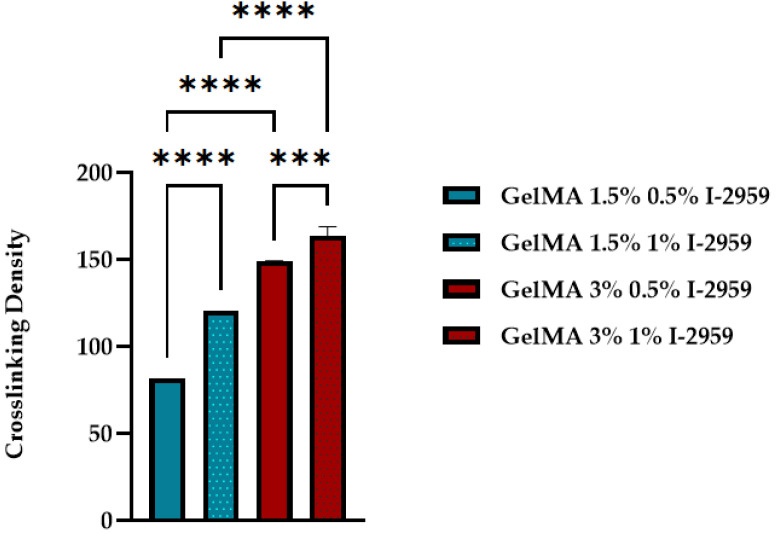
Crosslinking density of GelMA with different degrees of methacrylation and different concentrations of photoinitiator. Statistical significance: *** *p* < 0.0005; **** *p* < 0.0001.

Thus, a high concentration of photoinitiator used in the synthesis led to better mechanical properties. This fact is related to the formation of a more close-packed GelMA network resulting in the presence of a higher concentration of initiator. Figure 12 presents the statistical analysis of the crosslinking density for GelMA with two degrees of methacrylation and two different concentrations of photoinitiator.

**Figure 13:**
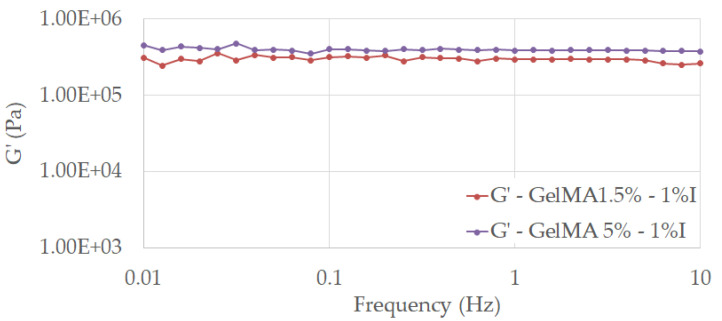
Storage modulus for the crosslinked hydrogels based on 1.5% GelMA with 1% I and 5% GelMA with 1% I as functions of frequency measured at 25 °C under a stress of 5 Pa.

**Figure 14:**
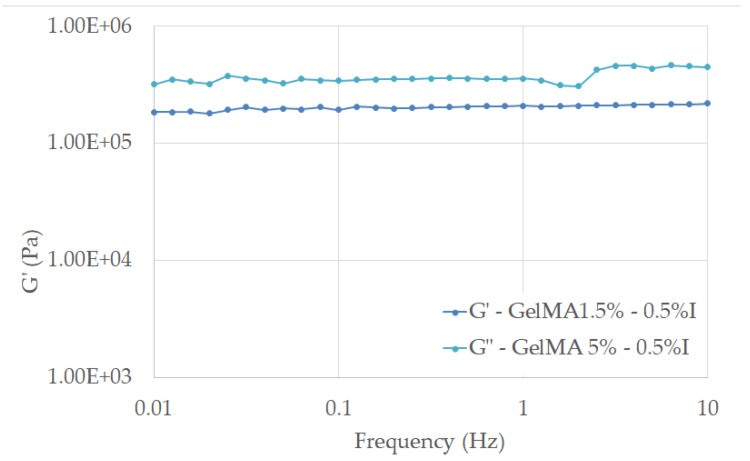
Storage modulus of crosslinked hydrogels based on 1.5% GelMA with 0.5% I and 5% GelMA with 0.5% I as functions of frequency measured at 25 °C under a stress of 5 Pa.

From the rheological tests, we can observe that all tested samples exhibited a dominant elastic behavior, with G’ > G”, almost independent of the frequency value, stated for the stability of the crosslinked hydrogels in the considered frequency range (Figure 13 and Figure 14). 

The results proved that the concentration of the photo initiator and methacrylation degree of GelMA are of great importance when we target the improvement in mechanical properties of a material.

Therefore, the higher methacrylation degree and concentration of photoinitiator used in the synthesis led to an improved mechanical behavior of GelMA. This fact is related to the formation of a more close-packed GelMA network as a consequence of the crosslinking reaction between more methacryloyl groups and initiation centers, as the calculated values of cross-linking density υ indicated (Figure 12). All these results are in good agreement with other studies conducted on hydrogels and GelMA hydrogels [29,30].

The computed values show that the crosslinking density is influenced by both the methacrylation degree (1.5% and 5%) of the modified protein and by the amount of initiator introduced in the system (0.5% and 1% Irgacure).

## 4. Conclusions

The focus of this research was to develop printable inks that could be used in regenerative medicine. Gelatin was chosen as the raw material because of its properties like: Biocompatibility, biodegradability, good mechanical properties. In order to improve the stability and mechanical properties, gelatin was modified with methacrylic anhydride. The ^1^H-NMR investigation highlighted the functionalization of gelatin with methacrylate functional groups that were grafted onto its backbone.

GelMA hydrogel inks with different GelMA concentrations and degrees of methacrylation were obtained and tested for printability. Printability studies showed that the concentration of GelMA but also photocrosslinker concentration in the precursor ink were key parameters for the obtaining of scaffolds with optimal integrity and structural geometry, but also with good mechanical properties. SEM analysis highlighted the highly porous morphology of GelMA-based scaffolds, a property that allows cell to attach, spread, and proliferate in order to achieve tissue regeneration. Thus, depending on the final application of the scaffold, the properties of the printing ink, but also the morphology of the final scaffolds, could be tailored with the help of the methacrylation degree, precursor, and photocrosslinker concentrations used in the formulation of the printing ink.

According to the obtained results, the GelMA hydrogel inks can be potential biomaterials for the development of 3D-printed GelMA-based scaffolds intended for biomedical application in the field of tissue engineering.

## Figures and Tables

**Table 1:** GelMA composition and methacrylation degree.

Sample	* G (g)	** MA (mL)	G:MA (*w*/*v*)	Methacrylation Degree (%)
***GelMA 1.5%***	5.00	0.75	6.66:1.00	63.00%
***GelMA 2.5%***	5.00	1.25	4.00:1.00	64.00%
***GelMA 5%***	5.00	2.5	2.00:1.00	66.00%

* Gelatin; ** Methacrylic anhydride. The methacrylation degree was calculated using Equation (1).

**Table 2:** The bioink preparation for 3D printing.

GelMA * Concentration (%)	10						20						
*GelMA methacrylation degree (%)*	63%		64%		66%		63%		64%		66%		
*I-2959 (%)*	0.5	1	0.5	1	0.5	1	0.5	1	0.5	1	0.5	1	

* GelMA-Gelatin Methacryloyl.

## Data Availability

The data presented in this study are available on request from the corresponding author.

## References

[1] Aldana A., Malatto L., Rehman M., Boccaccini A., Abraham G. (2019). Fabrication of Gelatin Methacrylate (GelMA) Scaffolds with Nano- and Micro-Topographical and Morphological Features. Nanomaterials.

[2] Neerajha N., Agnes D.-B., Erdem K., Pinar Z., Nihal E.V. (2018). Enabling personalized implant and controllable biosystem development through 3D printing. Biotechnol. Adv..

[3] Amol A.P., Gabriel S., Ido C., Liraz L., Joshua A.J., Seyed R.T., Nam-Joon C., Shlomo M. (2016). High-performance 3D printing of hydrogels by water-dispersible photoinitiator nanoparticles. Appl. Sci. Eng..

[4] Yin J., Yan M., Wang Y., Fu J., Suo H. (2018). 3D bioprinting of low concentration cell-laden gelatin methacrylate (GelMA) bioinks with two-step crosslinking strategy. ACS Appl. Mater. Interfaces.

[5] Echave M.C., Sánchez P., Pedraz J.L., Orive G. (2017). Progress of gelatin-based 3D approaches for bone regeneration. J. Drug Deliv. Sci. Technol..

[6] Sourbh T., Penny P.G., Messai A.M., Sigitas T., Vijay Kumar T. (2017). Recent progress in gelatin hydrogel nanocomposites for water purification and beyond. Vacuum.

[7] Lee J., Park C.H., Kim C.S. (2018). Microcylinder-laden gelatin-based bioink engineered for 3D bioprinting. Mater. Lett..

[8] Ward A.G. (1955). The Chemical Structure and Physical Properties of Gelatin. J. Photogr. Sci..

[9] Wang F., Li Y., Shen Y., Wang A., Wang S., Xie T. (2013). The functions and applications of RGD in tumor therapy and tissue engineering. Int. J. Mol. Sci..

[10] Na K., Shin S., Lee H., Shin D., Baek J., Kwak H., Park M., Shin J., Hyun J. (2018). Effect of solution viscosity on retardation of cell sedimentation in DLP 3D printing of gelatin methacrylate/silk fibroin bioink. J. Ind. Eng. Chem..

[11] Osama M., George M., Shawn D.W. (2019). Modified gelatin nanoparticles for gene delivery. Int. J. Pharm..

[12] Hoang T.T.T., Lee J.S., Lee Y., Park K.M., Park K.D. (2016). Enhanced cellular activity in gelatin-poly(ethylene glycol) hydrogels without compromising gel stiffness. Macromol. Biosci..

[13] Santoro M., Tatara A.M., Mikos A.G. (2014). Gelatin carriers for drug and cell delivery in tissue engineering. J. Control Release.

[14] Ding H., Illsley N.P., Chang R.C. (2019). 3D Bioprinted GelMA Based Models for the Study of Trophoblast Cell Invasion. Sci. Rep..

[15] Vandooren J., Van Den Steen P.E., Opdenakker G. (2013). Biochemistry and molecular biology of gelatinase B or matrix metalloproteinase-9 (MMP-9): The next decade. Crit. Rev. Biochem. Mol. Biol..

[16] Xiao S., Zhao T., Wang J., Wang C., Du J., Ying L., Lin J., Zhang C., Hu W., Wang L. (2019). Gelatin Methacrylate (GelMA)-Based Hydrogels for Cell Transplantation: An Effective Strategy for Tissue Engineering. Stem Cell Rev. Rep..

[17] Ye W., Li H., Yu K., Xie C., Wang P., Zheng Y., Zhang P., Xiu J., Tang Y., Zhang F. (2020). 3D printing of gelatin methacrylatebased nerve guidance conduits with multiple channels. Mater. Des..

[18] Liu J., Li L., Suo H., Yan M., Yin J., Fu J. (2019). 3D printing of biomimetic multi-layered GelMA/nHA scaffold for osteochondral defect repair. Mater. Des..

[19] Kilic Bektas C., Hasirci V. (2020). Cell loaded 3D bioprinted GelMA hydrogels for corneal stroma engineering. Biomater. Sci..

[20] Ismail H.M., Zamani S., Elrayess M.A., Kafienah W., Younes H.M. (2018). New Three-Dimensional Poly(decanediol-co-tricarballylate) Elastomeric Fibrous Mesh Fabricated by Photoreactive Electrospinning for Cardiac Tissue Engineering Applications. Polymers.

[21] Sun M., Sun X., Wang Z., Guo S., Yu G., Yang H. (2018). Synthesis and Properties of Gelatin Methacryloyl (GelMA) Hydrogels and Their Recent Applications in Load-Bearing Tissue. Polymers.

[22] Bigia A., Cojazzib G., Panzavoltaa S., Roveria N., Rubinia K. (2002). Stabilization of gelatin films by crosslinking with genipin. Biomaterials.

[23] Sheppard S.E., Elliott F.A. (1918). The Reticulation of Gelatine. J. Ind. Eng. Chem..

[24] Kim P., Yuan A., Nam K.H., Jiao A., Kim D.H. (2014). Fabrication of poly(ethylene glycol): Gelatin methacrylate composite nanostructures with tunable stiffness and degradation for vascular tissue engineering. Biofabrication.

[25] Nichol J.W., Koshy S.T., Bae H., Hwang C.M., Yamanlar S., Khademhosseini A. (2010). Cell-laden microengineered gelatin methacrylate hydrogels. Biomaterials.

[26] Aubin H., Nichol J.W., Hutson C.B., Bae H., Sieminski A.L., Cropek D.M., Akhyari P., Khademhosseini A. (2010). Directed 3D cell alignment and elongation in microengineered hydrogels. Biomaterials.

[27] Yue K., Trujillo-de Santiago G., Alvarez M.M., Tamayol A., Annabi N., Khademhosseini A. (2015). Synthesis, properties, and biomedical applications of gelatin methacryloyl (GelMA) hydrogels. Biomaterials.

[28] Credin C., Biella S., de Marco C., Levi M., Suriano R., Turri S. (2014). Fine tuning and measurement of mechanical properties of crosslinked hyaluronic acid hydrogels as biomimetic scaffold coating in regenerative medicine. J. Mech. Behav. Biomed. Mater..

[29] Shie M.-Y., Lee J., Ho C.-C., Yen S., Ng H.Y., Chen Y.-W. (2020). Effects of Gelatin Methacrylate Bio-ink Concentration on Mechano-Physical Properties and Human Dermal Fibroblast Behavior. Polymers.

[30] Fischer M., Maitz M.F., Werner C. (2018). Hemocompatibility of Biomaterials for Clinical Applications: Blood-Biomaterials Interactions.

[31] Kamel R., Ghazi B.M., Cyril J.F.K., Laura S.-G., Mouna K., Franck C., Solenne F., Michel L., Stéphane D., Elmira A.-T. (2017). Synthesis and Characterization of Nanofunctionalized Gelatin Methacrylate Hydrogels. Int. J. Mol. Sci..

[32] Agilent Technologies, Inc. (2013). Nano Indenter® G200 user manual.

[33] Bae H.L., Nathaniel L., Li Y.S., Pei QL Lay P.T. (2016). Synthesis and Characterization of Types A and B Gelatin Methacryloyl for Bioink Applications. Materials.

[34] Azami M., Ali S.I., Seyed A.P. (2010). Synthesis and characterization of a laminated hydroxyapatite/gelatin nanocomposite scaffold with controlled pore structure for bone tissue engineering. Int. J. Artif. Organs.

[35] Hossan M.J., Gafurb M.A., Kadirb M.R., Karim M.M. (2014). Preparation and characterization of gelatin-hydroxyapatite composite for bone tissue engineering. Int. J. Eng. Technol..

[36] Stephen R.M., Schilstra J.M. (2008). Circular Dichroism and Its Application to the Study of Biomolecules. Methods Cell Biol..

[37] Banerjee B., Misra G., Ashraf M.T. (2019). Circular Dichroism Data Processing Handbook for Complex Biological Data Sources.

[38] Donald A.M. (2001). Encyclopedia of Materials: Science and Technology. Guide-Wave Optical Communications: Materials.

[39] Kelly S.M., Price N.C. (2000). The use of circular dichroism in the investigation of protein structure and function. Curr. Prot. Pept. Sci..

[40] Ali M.S., Anjum K., Khan J.M., Khan R.H., Kabir ud D. (2011). Complexation Behavior of Gelatin with Amphiphilic Drug Imipramine Hydrochloride as Studied by Conductimetry, Surface Tensiometry and Circular Dichroism Studies Colloids. Surf. B Biointerfaces.

[41] Li Y., Du Z., Li G. (2008). Comparison of dynamic denaturation temperature of collagen with its static denaturation temperature and the configuration characteristics in collagen denaturation processes. Thermochim. Acta.

[42] Zhu M., Wang Y., Ferracci G., Zheng J., Cho N.-J., Lee B.H. (2019). Gelatin methacryloyl and its hydrogels with an exceptional degree of controllability and batchto-batch consistency. Sci. Rep..

[43] Barbara J.K., Debby G., Antoine J.W.P.R., Jos M., Ferry P.W.M. (2016). Gelatin-Methacryloyl Hydrogels: Towards Biofabrication-BasedTissue Repair. Trends Biotechnol..

[44] Luiz E.B., Juliana C.C., Vijayan M.A., Nupura S.B., Wesleyan A.A., Pinar Z., Nihal E.V., Amir M.G., Mehmet R.D., Ali K. (2014). Direct-write bioprinting of cell-laden methacrylated gelatin. Biofabrication.

[45] Diasa J.R., Baptista-Silvae S., Oliveiraa C.M.T., Sousaa A., Oliveirae A.L., Bártoloh P.J., Granjaa P.L. (2017). In situ crosslinked electrospun gelatin nanofibers for skin regeneration. Eur. Polym. J..

[46] Rauker J. (2013). Measuring Young’s Modulus of 20LP10L20-LLA40 Microspheres and Gelatin-Methacrylamide (GelMA) Hydrogel Using Nanoindentation. Master’s Thesis.

